# Therapeutic Potential of Alpha-Lipoic Acid in Viral Infections, including COVID-19

**DOI:** 10.3390/antiox10081294

**Published:** 2021-08-16

**Authors:** Stela Dragomanova, Simona Miteva, Ferdinando Nicoletti, Katia Mangano, Paolo Fagone, Salvatore Pricoco, Hristian Staykov, Lyubka Tancheva

**Affiliations:** 1 Department of Pharmacology, Toxicology and Pharmacotherapy, Faculty of Pharmacy, Medical University, 9002 Varna, Bulgaria; stela_dragomanova@abv.bg; 2 Department of Behavior Neurobiology, Institute of Neurobiology, Bulgarian Academy of Sciences, 1113 Sofia, Bulgaria; saalexandrova@gmail.com (S.M.); lyubkatancheva@gmail.com (L.T.); 3 Department of Biomedical and Biotechnological Sciences, University of Catania, Via S. Sofia 89, 95123 Catania, Italy; kmangano@unict.it (K.M.); paolofagone@yahoo.it (P.F.); salvatore.pricoco@yahoo.it (S.P.); 4 Department of Pharmacology and toxicology, Medical University, Sofia, 2, Zdrave Str., 1431 Sofia, Bulgaria; drhristianstaykov@gmail.com

**Keywords:** alfa-lipoic acid, antioxidants, natural products, oxidative responses, viral infections

## Abstract

Oxidative stress (OS), resulting from a disrupted balance between reactive oxygen species (ROS) and protective antioxidants, is thought to play an important pathogenetic role in several diseases, including viral infections. Alpha-lipoic acid (LA) is one of the most-studied and used natural compounds, as it is endowed with a well-defined antioxidant and immunomodulatory profile. Owing to these properties, LA has been tested in several chronic immunoinflammatory conditions, such as diabetic neuropathy and metabolic syndrome. In addition, a pharmacological antiviral profile of LA is emerging, that has attracted attention on the possible use of this compound for the cotreatment of several viral infections. Here, we will review the emerging literature on the potential use of LA in viral infections, including COVID-19.

## 1. Introduction: Oxidative Stress in Physiology and Pathophysiology

The cellular response to stress involves, among other biological processes, the production of both reactive oxygen species (ROS) and reactive nitrogen species (RNS), which are the primary representants of the prooxidant system [1]. ROS are composed of superoxides and peroxides and are characterized by strong microbicidal activities. In addition, the tonal levels of ROS have a wide range of significant effects in physiological cellular signaling and in survival mechanisms. The production of RNS is initiated with the synthesis of nitric oxide (NO) catalyzed by nitric oxide synthase (NOS). RNS include NO, which is relatively unreactive, and its derivative, peroxynitrite (ONOO^−^), a powerful oxidant. NO are highly reactive compounds that diffuse freely across cell membranes that can synergize with ROS into phagosomes and exert highly toxic effects on internalized microorganisms [1].

The maintenance of physiologically low and beneficial ROS levels is preserved through the action of several endogenous antioxidants, e.g., tripeptide glutathione (GSH). Alterations in the redox system lead to pathological effects, such as protein misfolding, cell damage, and death. In particular, oxidative stress has been associated with multiple chronic diseases, including atherosclerosis, type 2 diabetes, chronic kidney disease, and vascular calcification. The redox system also plays a role in viral infections and how the immune system responds to them. Many lines of evidence suggest that marked signs of increased production of ROS accompany all respiratory viral infections and that the exuberant production of ROS, associated with a defective antioxidant response, may be implicated in more severe forms of viral morbidity [1,2].

## 2. The Redox System in Infectious Diseases and Viral Infections

The deregulated production of ROS accompanies several viral infections, including influenza viruses (IV) and human respiratory syncytial virus (HRSV) and human rhinovirus [3].

During IV infections, a substantial increase in oxidation products can be detected both in the blood and urine. In a similar manner, augmented levels of ROS and nitric oxide synthase 2 (iNOS) nitrotyrosine, which witness nitrosative stress, have been reported in the lung tissues of patients that died in fatal IV pandemics [3]. IV-infected mice and cell lines are also characterized by the augmented production of ROS and the disturbance of antioxidant defense and, therefore, represent suitable tools to study the abnormalities in redox homeostasis induced by the virus.

Augmented ROS production is also induced by other respiratory viruses, including HRSV in A549 airway cells and in various cell lines by Sendai virus (SeV). Consistently, augmented lipid peroxidation products and oxidized GSH were observed in the circulation of infants with HRSV-induced acute bronchiolitis. SeV also triggers nitrosative stress, entailing augmented iNOS expression, enhanced production of nitric oxide, and accumulation of nitroguanosine. In addition, a reduced antioxidant capacity, another marker of oxidative stress, has been shown in HRSV-infected infants, mice and cells. Reduced levels of antioxidant enzymes have been also reported in airway cells and in mice replicating the human metapneumovirus (HMPV). Finally, the HRSV and human rhinovirus (HRV) were shown to induce the production of ROS in airway cells by enhancing the O2^•−^ production and by depleting the intracellular GSH [3].

Hepatitis C virus (HCV) also promotes oxidative stress and ROS production. Interestingly, the pharmacological induction of oxidative stress with Auranofin, a well-known prooxidant drug, markedly increased the viral RNA titer, thus indicating that a prooxidant state may favor the reactivation of HCV replication.

It has also been reported that three viral proteins of the hepatitis B virus (HBV), the surface antigens HBx, HBsAg, and the core antigen HBcAg induce ROS production.

The increased production of ROS, mediated by the envelope protein gp120 and Tat proteins, has also been reported during human immunodeficiency virus (HIV) infection. In addition, the overproduction of ROS induced by NOX2 and NOX4 has been reported in HIV gp120-treated astrocytes [4].

The level of oxidative stress dictates the immune response to viruses. For example, the oxidative stress observed during in vitro and in vivo Dengue virus (DENV) infections regulates the production of inflammatory cytokines, and the alteration in the redox state has been correlated with the disease severity. Moreover, ROS production controls the antiviral and apoptotic programs in DENV-infected human monocyte-derived dendritic cells (DC) [5].

In the presence of a well-balanced redox state, GSH is the main intracellular antioxidant that counteracts ROS via the thiol group of its cysteine that oxidizes to the disulfide form (GSSG), then is reduced back to the thiol form (GSH) by the GSH reductase [6,7,8]. Viral infections may lead to the intracellular depletion of GSH, as is the case for IV infection, where the reduction in GSH is required for the folding and maturation of viral glycoprotein haemagglutinin (HA) and, therefore, for viral replication [5]. The activation of nuclear factor erythroid 2-related factor 2 (Nrf2) also controls the homeostasis of the intracellular redox state. The Nrf2-mediated response can be differentially modulated by the viruses, depending on the phase of the replicative cycle and type of virus. For instance, during IV infection, the virus-induced oxidative stress leads to Nrf2 nuclear translocation and to the overexpression of antioxidant enzymes, such as HO-1, that may ultimately help to protect the cells from the virus-induced cytopathic effect [9]. Moreover, it has been shown that knocking down Nrf2 in human nasal epithelial cells facilitates influenza virus entry, as well as its replication, and that the supplementation with Nrf2-activating antioxidants inhibits viral replication.

Along the same lines, HCV infection can activate the Nrf2/ARE pathway, enhancing the expression of antioxidant genes and protecting the cells against HCV-induced oxidative stress [10,11]. However, the HCV-dependent inhibition of Nrf2-target gene expression may also favor the activation of the autophagic pathway, which is useful for the viral particles release [12]. Moreover, a biphasic expression of the Nrf2 pathway has been reported during the acute and chronic phases of HCV infection, which is characterized by downregulation during the early phases of infection and upregulation during the chronic phase [13].

On the ground of the role of the redox system in viral infections, it has been proposed that a dyshomeostatic redox system could also be implicated in the pathogenesis and the clinical course of a COVID-19 infection. In particular, it has been proposed a crucial role of GSH in determining individual responsiveness to SARS-CoV-2 infection and the feasibility of using GSH as a means for the treatment and prevention of COVID-19 [14]. Indeed, COVID-19 patients with moderate and severe levels of illness have been shown to have lower levels of GSH and higher ROS levels than COVID-19 patients with a mild level of illness [14].

Additionally, it was observed that the high neutrophil-to-lymphocyte ratio observed in critically ill patients with COVID-19 is associated with excessive levels of ROS, which promote a cascade of biological events that, in turn, drive the pathological host responses [15,16].

In addition, the Nrf2 pathway is suppressed in severe forms of SARS-CoV-2 infection, and, accordingly, the pharmacological inducers of Nrf2 are able to inhibit viral replication, the associated inflammatory response, and the activation of the transmembrane protease serine 2 (TMPRSS2), which is required for priming of the viral spike (S) protein and the subsequent virus–host cell membrane fusion and cell entry. Thus, Nrf2 activation may represent a new potential therapeutic approach for the COVID-19 pandemic [17].

On the ground of the aforementioned evidence, it has been proposed that approaches tailored at counteracting virus-induced oxidative stress could represent a novel therapeutic avenue for the treatment of viral infections or for the reduction of viral pathogenicity. Along this line of research, we will presently review the multiple pharmacological and antioxidant properties of the natural compound LA that are attracting significant attention. We will review the possible use of LA or its pharmacologically modified derivatives in the prevention and treatment of viral infections, including SARS-CoV-2 infection.

## 3. Beneficial Effects of LA

**Figure 1:**
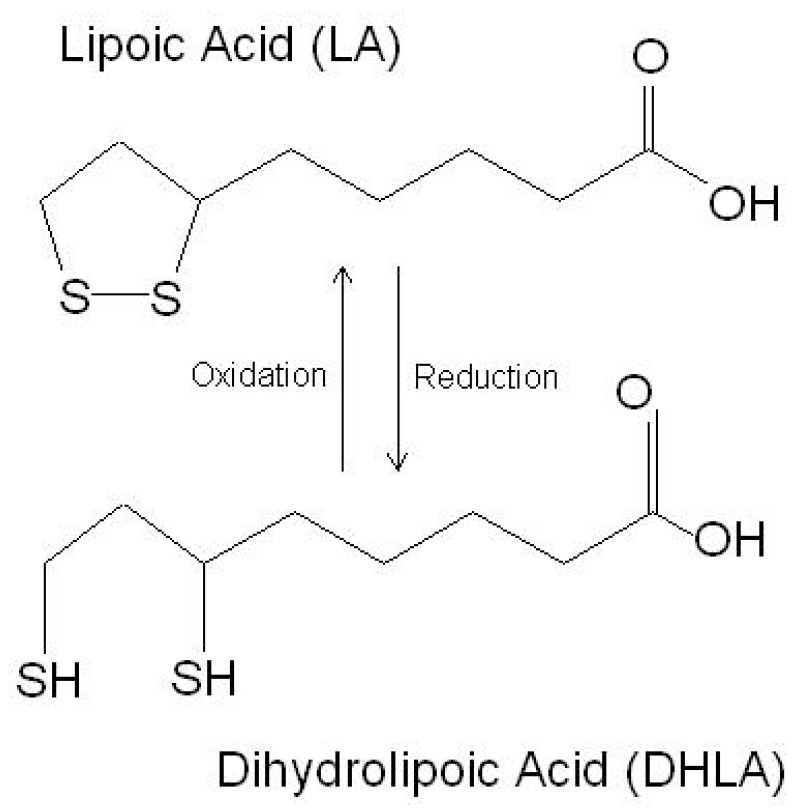
The chemical structure of lipoic acid and its active metabolite dihydrolipoic acid (https://www.robertbarrington.net/alpha-lipoic-acid-antioxidant/; posted on 3 January 2014 by Robert Barrington (accessed on 5 February 2021)).

LA is a powerful endogenous and exogenous antioxidant. It is a disulfide compound soluble in both water and oil. The reduced form of LA, dihydrolipoic acid, represents the active metabolite (Figure 1).

In the cell, LA increases the utilization of glucose in the mitochondria via supplying acetyl-CoA for the production of acetylcholine [18]. LA induces the activity of the enzymes responsible for the synthesis of GSH and other antioxidant enzymes and is a cofactor of several mitochondrial enzymes [19]. Hence, LA regulates several processes, such as nucleic acid synthesis and energy production, via the citric acid cycle [19].

LA is present in foods and supplements and possesses pleiotropic biological activities. As a free radical scavenger, LA increases the level of reduced GSH. Moreover, it regenerates vitamins C and E as part of the antioxidant defense system.

LA stimulates insulin production and improves glucose utilization, thus ameliorating the oxidative damage caused by hyperglycemia [20]. In addition, it reduces the insulin resistance and improves the insulin sensitivity and blood sugar control [21]. In patients with type 1 diabetes mellitus, LA leads to the downregulation of nuclear factor-kB in monocytes [22].

Additionally, LA has been shown to improve the symptoms of diabetic neuropathy, to reduce the risk of diabetic retinopathy [23,24,25], and to ameliorate diabetes-associated autonomic neuropathy [26].

Furthermore, LA improves endothelial dysfunction [27] and decreases the risks of heart attack and stroke [28].

LA can cross the blood–brain barrier (BBB) [18,29] and may alleviate the neuroinflammatory damage, preventing neuronal death, in models of Alzheimer’s disease and Parkinson’s disease [30,31,32,33,34]. Additionally, it significantly attenuates the inflammatory response in microglial cells, downregulating the production of proinflammatory cytokines, such as tumor necrosis factor-α (TNF-α) and IL-6, and other cytotoxic molecules, such as nitric oxide and ROS [35].

Maldonado-Rojas and colleagues (2011) performed an in silico screening of the molecular targets for LA, and, among several targets, leukotriene A4 hydrolase, voltage-gated potassium channel, alpha hydroxysteroid dehydrogenase, and epoxide hydrolase were found to be the most likely bound by LA. As these genes are involved in the pathogenesis of a number of diseases, this finding supports the potential use of LA in the treatment of cancer, diabetes, and neurological and cardiovascular disorders [36].

The proposed mechanisms of action for LA are summarized in Table 1.

## 4. Antioxidant Supplementation for the Treatment of Viral Infections

Antioxidant supplementation is expected to ameliorate the consequences of an infection, as oxidative stress is recognized as a major pathogenetic factor in several viral infections [3,68,69,70]. Indeed, many in vitro and in vivo studies have shown the positive effects of antioxidant therapies upon viral infections.

We have previously shown the effective targeting of the pathogenic mechanisms underlying influenza infections using antioxidant molecules, including vitamin E and vitamin C, flavonoids (i.e., rutin and quercetin), and polyphenols from *Geranium sanguineum* and *Punica granatum* [70,71,72,73,74,75]. In particular, a polyphenol-rich extract from the medicinal plant *Geranium sanguineum* L. administered to mice nasally challenged with lethal titers of the influenza A/Aichi/2/68 (H3N2) virus protected from mortality; prolonged the mean survival time; reduced the morphological alterations of the lungs; and regulated the ROS production and SOD, CAT, and LPO in the lungs [70,74]. Additionally, the flavonoids from rutin and quercetin restored oxidative damage in the lungs, blood, liver, and brain in IV-infected mice [75]. This is in line with the observations that quercetin is active against mosquito-borne diseases [76] and HCV [77]. Moreover, quercetin-3-*O*-d-glucuronide, quercetin-enriched lecithin formulations, and quercetin 7-rhamnoside have been shown to have beneficial effects against the porcine epidemic diarrhea virus and influenza A virus [78,79].

## 5. Effects of LA in Viral Infection

The accumulated in vitro and in vivo data demonstrated that LA can modulate the course of infection by affecting both the specific and nonspecific biochemical, virological, immunological, and neurological parameters associated with infections. In accordance, the beneficial effects of LA have been also observed in clinical trials.

### 5.1. Effects of LA on Influenza Virus Infection

Influenza viruses are RNA viruses of the Orthomyxoviridae family. Seasonal epidemics are associated with 3–5 million cases of severe illness each year and from 290- to 650-thousand deaths worldwide (https://www.who.int/news-room/fact-sheets/detail/influenza-(seasonal); accessed on 3 August 2021). Although safe and effective vaccines are available, their specificity is limited because of the diversity of the circulating influenza strains and the ability of the virus to acquire mutations. The treatment options for influenza infections are limited to inhibitors of neuraminidase and blockers of the M2 proton channel. In patients infected with IV, marked increases of oxidation byproducts are found, including 8-hydroxydeoxyguanosine, malondialdehyde, F2-isoprostane, 7-ketocholesterol, and 7β-hydroxycholesterol (reviewed in reference [3]). Additionally, elevated levels of sterol oxidation products are observed up to three months after IV clearance (reviewed in reference [3]). Finally, increased levels of ROS, as well as of nitric oxide synthase 2 (iNOS) and nitrotyrosine, are found in the lung tissues of patients that died from an IV infection (reviewed in reference [3]). Several reports have shown that NAC, ascorbic acid, and vitamin E have positive effects on IV infections by inhibiting viral replication and inflammation both in the cells and in the mice (reviewed in reference [3]). In an in vitro study, LA inhibited IV replication in MDCK cells and reduced the nuclear translocation of NF-κB. Additionally, the caspase-3 activity was remarkably inhibited and type I interferons (IFNs) were upregulated upon LA treatment [80]. Overall, the available data indicated that LA might be a potential anti-influenza agent, deserving further investigation.

### 5.2. Effects of LA in Herpes Infections

Herpes virus infection is widely spread, as estimates show that 80% of people worldwide are carriers of at least one strain. Nine herpesvirus types are known to primarily infect humans: herpes simplex viruses 1 and 2 (HSV-1 and HSV-2), varicella zoster virus, Epstein–Barr virus, human cytomegalovirus human herpesvirus 6A and 6B (HHV-6A and HHV-6B), human herpesvirus 7 (HHV-7), and Kaposi’s sarcoma-associated herpesvirus (KSHV) [81]

LA has been recognized for its potential to control herpes virus infections in a patented invention—a formulation for external use containing different naturally occurring substances, together with LA. The expected beneficial effect is to narrow the period of inflammation, allowing the affected tissues to be restored (US patent application No.: US 2011/0229584A1).

The neuroprotective properties of LA could potentially find their role in post-herpetic neuralgia, following shingles or Herpes zoster infection [29,34]. The chronic damage caused by the virus results in so-called post-herpetic neuralgia, could potentially be alleviated thanks to the neuroprotective properties of LA, that have been demonstrated in diabetic polyneuropathy—another condition related to peripheral nerve damage. In addition, by acting on the endothelial cells, LA improves the endoneural blood flow and favors the transport of nutrients to the compression site responsible for neuronal damage [27].

### 5.3. Effects of LA in Smallpox Infection

Smallpox was declared eradicated in 1980; however, in recent years, due to the potential use of this virus as a biological weapon, concerns have been raised for an improved method for the prevention and treatment of this disease. The currently available vaccines, which have not been updated since the eradication of smallpox, could lead to severe adverse reactions, and interest in drugs with good safety profiles is emerging. In addition to the prevention and treatment of vaccinia-related infections and variola infections, worth mentioning is the so-called monkey pox virus, which is endemic for Africa, although cases are also sporadically reported in the United States.

An in vitro study of LA and ethacrynic acid has shown that both substances have inhibitory effect towards virus growth, while they are relatively nontoxic to fibroblasts and epithelial cells [82]. The effect of LA and ethacrynic acid was dose-dependent and seemed to be related to the inhibition of the expression of VACV late genes, resulting in decreased levels of infectious virus progeny. The two molecules, however, did not seem to affect either VACV entry into the cell or viral DNA synthesis [82].

### 5.4. Effects of LA in Viral Hepatitis

Chronic viral hepatitis is the leading cause of liver fibrosis and cirrhosis. Replication of the virus in liver cells results in decreased levels of glutathione in hepatocytes. The viral load in hepatitis C is directly related to the glutathione levels [83]. Eventually, the oxidative stress leads to necrotic inflammation and the necrosis of liver cells. Increased lipid peroxidation leads to hepatic cytotoxicity, causing immune-mediated inflammation and fibrosis [84,85,86]. Supportive therapeutic effects of LA have been demonstrated in the clinical setting in patients with a Hepatitis C infection [87,88]. The LA antioxidant properties have been recognized as potential therapeutic effects based on its ability to exert anti-inflammatory, antifibrotic, and anti-TNF-α effects that produce restoration of the damaged areas of the liver, as well as its function, as demonstrated in HCV patients [87,88]. According to a clinical study with a small number of patients conducted by Berkson in 1999, therapy with the antioxidant LA, the hepatoprotector silymarin, and the element selenium, completely restores the liver function of patients, and the proposed combination allows avoiding liver transplantation [87]. The beneficial effect of the application of various antioxidants, including LA, was later confirmed in a clinical trial enrolling 50 patients with chronic hepatitis C infection [88]. Among them, 48% responded with improvement in at least one of the observed liver parameters, and a decrease in the viral load was observed in 25% of the patients.

### 5.5. Effects of LA in HIV Infection

A major pathogenetic factor in HIV infection is the induced oxidative stress, which causes apoptosis and a decrease in the number of CD4 + T cells, which may eventually lead to marked immunosuppression [89,90]. Increased oxidative reactions [89] and higher concentrations of hydrogen peroxide [91] and MDA [92], as well as suppression of the antioxidant protection response, with decreased activity of the antioxidant enzymes SOD, GPx, CAT, and thioredoxin, have been found in the plasma, lung mucosa, erythrocytes, and lymphocytes from patients with HIV infection [90,91,93].

The potential antiviral effects of LA were first proposed by Baur and colleagues in 1991 [94]. They observed that the addition of LA to the T-cell lines Jurkat, SupT1, and Molt-4 incubated with a wild-type HIV-1 isolate was associated with a dose-dependent inhibition of HIV-1-replication, a reduced cytopathic effect, and the inhibition of reverse transcriptase activity and plaque formation [94]. Moreover, LA synergized with zidovudine in inhibiting viral replication by affecting the reverse transcriptase enzyme [94].

In HIV patients, the administration of LA was associated with positive effects characterized by the stable restoration of the total blood GSH levels and lymphocyte function in comparison to the placebo group, where a progressive decline was observed [95].

In another clinical study, upon the oral administration of LA to 11 AIDS patients, an increase in the blood concentrations of ascorbic acid and total GSH and a decrease in the levels of lipid peroxidation, accompanied by a significant increase in the CD4/CD8 ratio and the number of CD4 + T cells in the plasma, was observed [96].

### 5.6. Effects of LA in COVID-19

The severity of COVID-19 seems to depend on the massive release of proinflammatory cytokines, including interleukin (IL)-1β, IL-2, IL-6, IL-7, IL-8, TNF-α, and chemokines such as CXC-chemokine ligand 10 (CXCL10) and CC-chemokine ligand 2 (CCL2), known as the “cytokine storm”. This is likely to promote a self-sustained inflammatory reaction that may ultimately lead to the development of respiratory and multiple organ failure, with a consequent shock and disseminated intravascular coagulation. Besides the respiratory system, SARS-CoV-2 affects other organs, including the heart, kidneys, and the central nervous system. Indeed, COVID-19 patients may have elevated serum creatinine levels, proteinuria, and hematuria and can develop neurological symptoms, such as headaches, parkinsonism, and anosmia. In addition, a thrombogenic diathesis, characterized by the elevation of D-dimer and other procoagulant parameters, can be observed and may be responsible for some lethal cases of COVID-19 [97,98,99,100,101,102,103].

Several lines of evidence suggest the potential beneficial effects of LA in SARS-CoV-2 infection. Indeed, the affinity of SARS-CoV-2 for the ACE2 receptor is augmented by the oxidation of the cysteine residues in the S protein (RBD) of the virus and the cell membrane ACE2 (peptidase domain). Hence, the imbalance of redox homeostasis could represent a major factor favoring SARS-CoV-2 infection [104]. Therefore, it is likely that the cell entry of SARS-CoV-2 could be impaired in the presence of the LA treatment. In addition, by reducing the ROS levels, LA can exert an anti-inflammatory effect, decreasing the proinflammatory cytokine secretion. Additionally, by maintaining an endogen high GSH/GSSG ratio, LA may prevent severe forms of COVID-19. The GSH levels decrease in COVID-19 due to a deficient nuclear translocation of Nrf2 [105]. LA promotes GSH synthesis by increasing the cellular cysteine uptake, a rate-limiting factor for GSH synthesis, and by activating the Nf2-ARE signaling pathways, with an increased activity of glutamate–cysteine ligase, the rate-limiting enzyme in GSH production [105]. Finally, given the key contribution of mitochondrial dysfunction in COVID-19 pathogenesis [106], LA is thought to be able to reduce mitochondrial oxidative damage, as it functions as a cofactor for α-ketoglutarate dehydrogenase, a sensor of the mitochondrial redox status that regulates the mitochondrial cytochrome c release and cell death. A report, still in the phase of scientific evaluation, shows the results from a randomized, single-blind trial performed at Jin Yin Tan Hospital, Wuhan, China that enrolled 17 patients with critically ill COVID-19, who were assigned in a 1:1 ratio to receive either LA (1200 mg/d, intravenous) once-daily plus the standard care or the standard care plus an equal volume of saline infusion (placebo) for 7 days. In accordance with the above-mentioned hypotheses, LA was associated with a lower SOFA score increase with respect to the baseline, and a two-fold higher mortality rate was observed in the placebo group, as compared to the LA group. Although the results are encouraging, larger cohorts of patients are needed to validate the efficacy of LA in COVID-19 [107].

## 6. Conclusions

It has been proposed that the pharmacological approaches aimed at counteracting virus-induced oxidative stress could represent a feasible therapeutic avenue for the treatment of viral infections and to reduce the severity of their symptoms. In this regard, the natural compound LA is attracting significant attention, as LA can modulate the course of an infection by acting as a free radical scavenger, dampening excessive inflammation and exerting specific antiviral actions. The pharmacological properties of LA also make it a potential candidate for the treatment of SARS-CoV-2 infection.

## Figures and Tables

**Table 1:** Proposed mechanisms of action of LA.

Mechanism of Action	References
Scavenging reactive oxygen species	[37,38,39]
Regeneration other endogenous antioxidants (e.g., vitamins C and E)	[40]
Chelation of redox-active metals	[41,42,43,44]
Induction of endogenous antioxidants (e.g., ascorbate, GSH)	[45,46,47,48,49,50]
Induction of the Nrf2/ARE pathway	[51,52,53,54]
Inhibition of NF-κB activation	[55]
Activation of:	--
- PKC	[56]
- Erk1/2	[57,58]
- p38 MAPK	[59]
- PI3K	[59]
- Akt	[59,60,61,62]
- Inhibition of:	--
- PTEN	[60]
- PP2A	[60]
- PTP1B	[63]
Activation of insulin receptor by direct binding	[64]
Activation of AMP-activated protein kinase (AMPK)	[65,66,67]
